# Deep Concatenated Residual Networks for Improving Quality of Halftoning-Based BTC Decoded Image

**DOI:** 10.3390/jimaging7020013

**Published:** 2021-01-25

**Authors:** Heri Prasetyo, Alim Wicaksono Hari Prayuda, Chih-Hsien Hsia, Jing-Ming Guo

**Affiliations:** 1 Department of Informatics, Universitas Sebelas Maret, Surakarta 57126, Indonesia; wicayudha.wy@gmail.com; 2 Department of Computer Science and Information Engineering, National Ilan University, Yilan 260, Taiwan; 3 Department of Electrical Engineering, National Taiwan University of Science and Technology, Taipei 106335, Taiwan; jmguo@seed.net.tw

**Keywords:** block truncation coding, convolutional neural networks, deep learning, halftoning, residual learning, image reconstruction

## Abstract

This paper presents a simple technique for improving the quality of the halftoning-based block truncation coding (H-BTC) decoded image. The H-BTC is an image compression technique inspired from typical block truncation coding (BTC). The H-BTC yields a better decoded image compared to that of the classical BTC scheme under human visual observation. However, the impulsive noise commonly appears on the H-BTC decoded image. It induces an unpleasant feeling while one observes this decoded image. Thus, the proposed method presented in this paper aims to suppress the occurring impulsive noise by exploiting a deep learning approach. This process can be regarded as an ill-posed inverse imaging problem, in which the solution candidates of a given problem can be extremely huge and undetermined. The proposed method utilizes the convolutional neural networks (CNN) and residual learning frameworks to solve the aforementioned problem. These frameworks effectively reduce the impulsive noise occurrence, and at the same time, it improves the quality of H-BTC decoded images. The experimental results show the effectiveness of the proposed method in terms of subjective and objective measurements.

## 1. Introduction

The block truncation coding (BTC) is a type of lossy image compression technique under the block-wise processing manner [1]. In the encoding process, an input image is firstly divided into a set of image blocks, in which one block is non-overlapping with the other blocks. Each image block is processed individually to yield two extreme quantizers, namely high and low mean values, and a binary image. The high and low mean values are computed in such a way using the average value (mean value) and standard deviation on each processed image block. The magnitude of high mean value is higher compared to the low mean value. These two means keep the image block statistics unchanged. The required bit to represent each image block can be significantly reduced using this strategy, while the underlying statistical property of an image block (the mean value and standard deviation) can be still maintained. In the decoding process, a pixel value of a binary image is simply replaced with high or low mean value. From this point of view, the BTC compression is very easy to implement. However, the false contour and blocking artifacts often destroy the quality of the BTC decoded image. These artifacts are more noticeable while the size of the image block is increased.

On other hand, the halftoning-based block truncation coding (H-BTC) method beats the performance of the classical BTC by introducing the digital halftoning technique to generate visual illusion on its binary image [2,3,4]. The H-BTC replaces the binary image used in the BTC technique with the common digital halftone image obtained from the ordered dither, error diffusion, and dot diffusion. While these halftoning techniques are integrated with H-BTC, we pronounce as ordered dither block truncation coding (ODBTC), error diffusion block truncation coding (EDBTC), and dot diffused block truncation coding (DDBTC). As reported in literature, the H-BTC method and its variants yield a better quality of decoded image and overcome the artifact problems that occurred at the BTC technique. Even though the H-BTC method and its variants successfully compress an image with better quality compared to that of the classical BTC technique, they generally produce a decoded image with poor quality due to the occurrence of impulsive noise. This impulsive noise is more perceivable and noticeable while the H-BTC compression is applied to the color image over a large image block. Some solutions have been offered to overcome these shortcomings such as in [5,6]. The wavelet-based approach [5] separates the occurred noise into the high-frequency sub-band, while the image information including detail, edge, and intrinsic geometric property are located in the low-frequency sub-band. By modifying the high and low-frequency sub-bands, this simple technique successfully eliminates the occurred impulsive noise. However, the reconstructed image looks blurry and produces some checkerboard artifacts. In different directions, the fast vector quantization (VQ) [6] conducts the H-BTC image reconstruction by substituting each image patch of the H-BTC decoded image with the similar image patch from a trained codebook. In this technique, the trained codebook is generated from a set of clean images, thus, each codeword is a noise-free image patch. It is no wonder that the VQ-based approach gives better performance compared to the wavelet-based scheme. However, the reconstructed image is still blurry with an unpleasant appearance. The developed methods in [5,6] share a similar idea, i.e., performing the inverse halftoning to suppress the occurred impulsive noise in the H-BTC decoded image. The inverse halftoning mainly performs the restoration from the halftone image into its continuous-tone version. However, this task becomes non-trivial due to many-to-one nature on the quantization process of halftoning computation. Many different input levels are quantized into one value, i.e., black or white tone. Thus, the inverse halftoning has no unique solution.

The deep learning frameworks have attracted so much attention in recent years due to its outstanding performance in image processing and computer vision tasks [7,8,9,10,11,12,13,14,15,16,17,18,19,20,21]. The convolutional neural networks (CNN) and residual [7] learnings are the most well known amongst the other deep learning techniques. The CNN involves several convolution operations, activation function, and image batch normalization in the learning process. Whereas, the residual networks (ResNets) learn an end-to-end mapping between the input and targeted output image based on the residual information contained in a series of convolutional layers. The CNN, ResNets, and its variants have been reported in literature [7,8,9,10,11,12,13,14,15,16,17,18,19,20,21] to yield an excellent performance in the image retrieval, image super resolution, image denoising, etc. In recent years, some efforts have been devoted to further improve the performance and effectiveness of CNN methods by considering the non-local self-similarity information. An example are the neural nearest neighbor networks [18]. This scheme employs the non-local self-similarity as a building block to perform image denoising. It overcomes the limitation of the K-nearest neighbor method by offering relaxation for neighbor selection. Whereas, the method in [19] conducts an image restoration with non-local recurrent networks by learning the non-local information and adjacent recurrent states. It computes the deep features from neighborhood information of a given input image. The method in [20] combines the CNN and non-local self-similarity to create the graph convolutional networks for the image denoising task. As reported in literature [18,19,20], the CNN-based method with non-local self-similarity gives better performance compared to that of the original CNN-based scheme. The U-Nets has also been reported as an outstanding performance in image segmentation [21]. It is built on the CNN and residual learning frameworks.

Based on these observations, we propose a new technique for improving the quality of an H-BTC decoded image using a deep learning framework. This technique learns much information from a set of training images to investigate the ill-posed problem and the many-to-one nature of inverse halftoning schemes. This learning process produces a model to infer and suppress the impulsive noise for improving the quality of the H-BTC decoded image. Herein, we employ the CNN and residual learning with end-to-end mapping ability. The proposed networks receive the H-BTC decoded image and produce the improved quality of this decoded image.

The rest of this paper is organized as follows. Section 2 briefly discusses the H-BTC image compression and related works for improving the quality of the decoded image. Section 3 presents the proposed deep learning framework for H-BTC image reconstruction. Section 4 reports the experimental results and findings. The conclusions are finally drawn in Section 5.

## 2. Related Works

This section briefly reviews the three H-BTC compression methods, namely ordered dither block truncation coding (ODBTC), error diffusion block truncation coding (EDBTC), and dot diffused block truncation coding (DDBTC). The two methods for improving the quality of H-BTC decoded image, i.e., wavelet-based approach and VQ based technique, are also presented in this section.

### 2.1. H-BTC Image Compression

**Figure 1:**
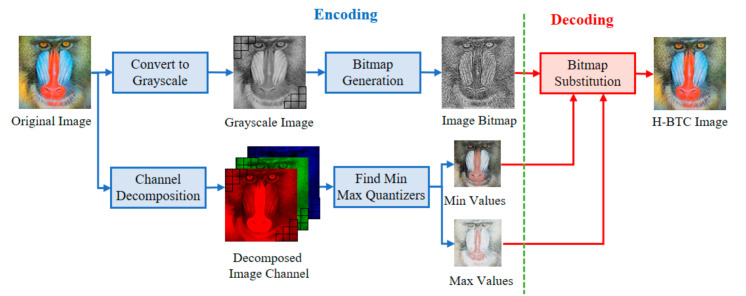
Schematic diagram of halftoning-based block truncation coding (H-BTC) image compression.

The variants of H-BTC image compression, i.e., ODBTC, EDBTC, and DDBTC, transform a continuous-tone image into another representation to reduce the required bit. Figure 1 illustrates the schematic diagram of H-BTC compression for color image regarded as continuous-tone. In this compression, a color image of size H×W is firstly divided into non-overlapping blocks, each of size M×N. Let $I\left(m,n\right)=\left\{x_{m,n}^{R},\;x_{m,n}^{G},\;x_{m,n}^{B}\right\}$ be an image block in color version, for m=1,2,…,M and n=1,2,…,N, where $x_{m,n}^{R}$, $x_{m,n}^{G}$, and $x_{m,n}^{B}$ denote the pixel values on red, green, and blue channels, respectively. Suppose that $\hat{I}\left(m,n\right)$ be the grayscale version of an image block $I\left(m,n\right)$. This image block $I\left(m,n\right)$ is further encoded into two extreme quantizers and a bitmap image based on the following function:

(1) $$H\left\{I\left(m,n\right)\right\}\Rightarrow \left\{q_{\min},\;q_{\max},\;b\left(m,n\right)\right\},$$

where $q_{\min}$ and $q_{\max}$ are two extreme quantization values, $b\left(m,n\right)$ is the binary or bitmap image of size M×N. These quantization values can be simply obtained by searching the minimum and maximum pixel values within the image block as follows:

(2) $$q_{\min}=\left\{\underset{\forall m,n}{\min}x_{m,n}^{R},\underset{\forall m,n}{\min}x_{m,n}^{G},\underset{\forall m,n}{\min}x_{m,n}^{B}\right\},$$

(3) $$q_{\max}=\left\{\underset{\forall m,n}{\max}x_{m,n}^{R},\;\underset{\forall m,n}{\max}x_{m,n}^{G},\underset{\forall m,n}{\max}x_{m,n}^{B}\right\}.$$

The ODBTC, EDBTC, and DDBTC compression employ the same extreme quantization values. The main difference between those methods is only on the bitmap image generation process. This bitmap image is generated from the grayscale version of an image block. The ODBTC utilizes a dither array, while the EDBTC uses the error kernel. Whereas, the DDBTC involves class rank and diffused weighting matrices.

Let $D\left(m,n\right)$ be a dither array of the same size with the image block, i.e., M×N. The dither array is generated by involving a set of training images and learning process. The optimum dither array is obtained by an iterative process by minimizing similarity error between the input image and its thresholded image after dithering process. Prospective readers are suggested to refer [2] for detailed explanation of dither array generation. Given a set of training images, the dither array is iteratively updated based on the similarity. The scaled versions of this dither array are pre-calculated and stored as a look-up table denoted with $\left\{D^{\left(0\right)},\;D^{\left(1\right)},\ldots ,D^{\left(255\right)}\right\}$. This look-up table significantly reduces the computational time on generating the ODBTC bitmap image [2]. Subsequently, the ODBTC encodes the grayscale version of the image block $\hat{I}\left(m,n\right)$ using the simple thresholding as follows:

(4) $$b\left(m,n\right)=\left\{\begin{array}{c} 0,\;\mathrm{if}\;\hat{I}\left(m,n\right)<D^{\left(k\right)}\left(m,n\right)+{\hat{x}}_{min}\\ 1,\;\mathrm{otherwise} \end{array}\right.,$$

where ${\hat{x}}_{min}$ denotes the minimum pixel value in the image block $\hat{I}\left(m,n\right)$, and $d=q_{\max}-q_{\min}$. In contrast, the EDBTC method generates the bitmap image by means of error kernel [3]. The Floyd–Steinberg kernel is very popular among the other kernels in the error diffusion halftoning. The generation of this error kernel can be traced back in [3]. Yet, the thresholding process for computing the EDBTC bitmap image is formally defined as:

(5) $$b\left(m,n\right)=\left\{\begin{array}{c} 0,\;\mathrm{if}\;v_{m,n}<\bar{x}\\ 1,\;\mathrm{otherwse} \end{array}\right.,$$

where $\bar{x}$ denotes the mean value over all pixels in the image block $\hat{I}\left(m,n\right)$. The EDBTC further diffuses the error value obtained from this thresholding operation into its neighboring pixels based on the error kernel value. This error diffusion process can be simply formulated as follows:

(6) $$v_{m,n}={\hat{x}}_{m,n}+x_{m,n}^{\prime },$$

(7) $$e_{m,n}=v_{m,n}-o_{m,n},$$

where $x_{m,n}^{\prime }=e_{m,n}*k$. The value of $o_{m,n}={\hat{x}}_{min}$ if $v_{i,j}<\bar{x}$, and $o_{m,n}={\hat{x}}_{max}$ for vice versa. Herein, the symbols $e_{m,n}$, $o_{m,n}$, k, and * denote the residual quantization error, intermediate value, the value of error kernel, and convolution operator, respectively. The symbols ${\hat{x}}_{min}$ and ${\hat{x}}_{max}$ are the maximum and maximum pixel values, respectively, found in $\hat{I}\left(m,n\right)$.

Whereas, the DDBTC combines the effectiveness of ordered dithering and error diffusion halftoning techniques to achieve outstanding performance [4]. The DDBTC utilizes the class and diffusion matrices. The class matrix is of the same size as the image block, which determines the pixel processing order, while the diffusion matrix contains information for distributing the residual quantization error into its neighborhood pixels. The process of DDBTC thresholding can be executed using the following strategy as formerly used in [4]:

(8) $$v_{m,n}={\hat{x}}_{m,n}+x_{m,n}^{\prime },\;\mathrm{where}\;x_{m,n}^{\prime }=\frac{e_{m,n}*d}{sum},$$

(9) $$sum=\sum_{k=-1}^{1}\sum_{l=-1}^{1}\left\{\begin{array}{c} 0\;\mathrm{if}\;c_{m+k,n+l}<c_{m,n}\;\\ h_{m,n}\;\mathrm{otherwise} \end{array}\right.,$$

where d, $c_{m,n}$, and sum indicate the diffusion matrix, coefficient value in class matrix, and the summation of diffused weights corresponding to those unprocessed pixels, respectively. The DDBTC performs an identical thresholding operation as used in the EDBTC for generating the bitmap image.

All H-BTC methods transmit two extreme quantization values and bitmap image into the decoder side. Yet, the decoder simply replaces the bitmap image of value 1 with the max quantizer value, and vice versa. The decoding process of H-BTC methods is performed using the following strategy:

(10) $$r\left(m,n\right)=\left\{\begin{array}{c} q_{\min},\;\mathrm{if}\;b\left(m,n\right)=0\\ q_{\max},\;\mathrm{otherwise}\; \end{array}\right.,$$

where r is the decoded pixel at position $\left(m,n\right)$ for m=1,2,…,M and n=1,2,…,N. This decoding process is very efficient, making it very suitable for real-time application.

**Figure 2:**
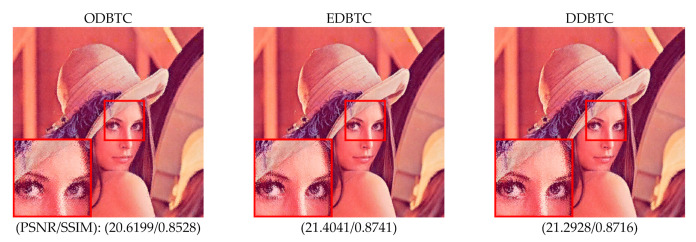

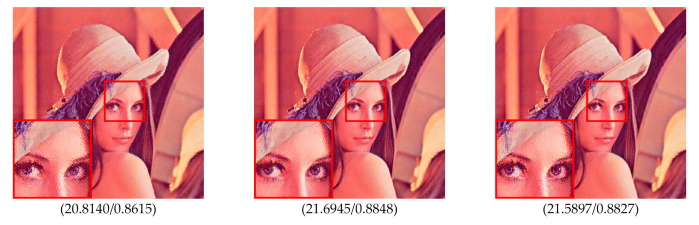
Visual comparisons of using single and three bitmap images on (first to last column) ordered dither block truncation coding (ODBTC), error diffusion block truncation coding (EDBTC), and dot diffused block truncation coding (DDBTC). The first and second rows are using single and three bitmap images, respectively.

In this paper, a single bitmap image is utilized in the ODBTC, EDBTC, and DDBTC. As illustrated in Figure 1, we firstly need to compute the grayscale version of a given color image. Subsequently, the image thresholding is performed to convert this grayscale image into a bitmap image. This process effectively reduces the computational burden in the encoding side. In addition, the required bit for storing a single bitmap image is lower compared to that of keeping three bitmap images. But, the quality of H-BTC decoded image using a single bitmap image is slightly inferior in comparison to the three bitmap images. Figure 2 shows visual comparisons of using a single and three bitmap images over ODBTC, EDBTC, and DDBTC compression. The first and second rows are using single and three bitmap images, respectively. This figure demonstrates that quality of the decoded image using a single bitmap image is degraded compared to employing three bitmap images. It will be more challenging if we are able to improve the quality of the H-BTC decoded image using a single bitmap image.

### 2.2. Wavelet-Based H-BTC Image Reconstruction

**Figure 3:**
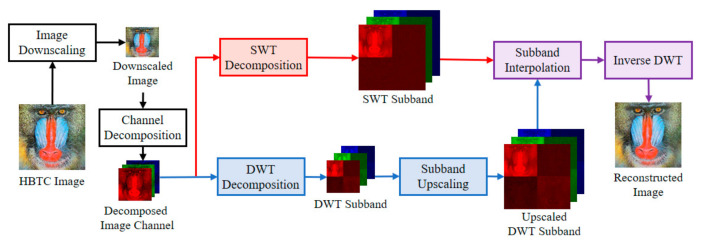
Schematic diagram of wavelet-based H-BTC image reconstruction [5].

The quality of the H-BTC decoded image is less satisfied for human vision due to impulsive noise occurrence. Thus, the wavelet-based method [5] tries to improve the quality of this decoded image quality based on the fact of noise placement. Commonly, the image noise remains on high-frequency subbands of the wavelet transformed image, while image information lies on low frequency subbands. More precisely, the H-BTC reconstructed image is obtained from the lowpass filtered and downsampled version of an input image [5]. Figure 3 draws a schematic diagram of wavelet-based image reconstruction [5]. Suppose that the downsampled version of the H-BTC decoded image is denoted as $I^{↓\times 2}$. This image is of size M×N, where $M=\frac{H}{2}$ and $N=\frac{W}{2}$, respectively. Herein, the discrete wavelet transform (DWT) [22,23] is utilized to decompose an image $I^{↓\times 2}$ as follows:

(11) $$J\left\{I^{↓\times 2}\right\}\Rightarrow \left\{I_{\theta }^{↓\times 2}|\;\theta =\left(LL,\;LH,\;HL,\;HH\right)\right\},$$

where θ and $J\left\{\cdot \right\}$ denote the DWT image subbands and DWT operator, respectively. This decomposition process yields a transformed image $I_{\theta }^{↓\times 2}$ of size $\frac{M}{2}\times \frac{N}{2}$. Subsequently, an image interpolation process with upscaling factor 2 is applied to $I_{\theta }^{↓\times 2}$. Yet, this process produces an upscaled image $I_{\theta }^{↑\times 2}$ of size M×N.

At the same time, the stationary wavelet transform (SWT) decomposes an image $I^{↓\times 2}$ into the following subbands:

(12) $$J^{*}\left\{I^{↓\times 2}\right\}\Rightarrow \left\{I_{\theta^{*}}^{↓\times 2}|\;\theta^{*}=\left(LL,\;LH,\;HL,\;HH\right)\right\},$$

where $\theta^{*}$ and $J^{*}\left\{\cdot \right\}$ denote the SWT image subbands and SWT operator, respectively. Herein, each image subband $I_{\theta^{*}}^{↓\times 2}$ is of size M×N. From this stage, we obtain paired image subbands with the same size, i.e., the upscaled DWT and SWT subbands. Performing the addition process between these paired subbands effectively eliminates the impulsive noise. The addition process can be denoted as follows:

(13) $${\tilde{I}}_{\theta }^{↓\times 2}=\alpha_{\theta }I_{\theta }^{↑\times 2}+\left(1-\alpha_{\theta }\right)I_{\theta^{*}}^{↓\times 2},$$

where $\alpha_{\theta }$ denotes a specific constant controlling the percentage contribution of upscaled DWT and SWT image subbands. This addition process is applied to all subbands θ and $\theta^{*}$. Symbol ${\tilde{I}}_{\theta }^{↓\times 2}$ indicates the modified image subbands. Finally, the following process is executed for overall ${\tilde{I}}_{\theta }^{↓\times 2}$:

(14) $$\hat{I}⇐J^{-1}\left\{{\tilde{I}}_{\theta }^{↓\times 2}|\;\theta =\left(LL,\;LH,\;HL,\;HH\right)\right\},$$

where $\hat{I}$ is the H-BTC reconstructed image, and $J^{-1}\left\{\cdot \right\}$ indicates the inverse DWT operator. From this process, one obtains the H-BTC reconstructed image of the same size with the original H-BTC decoded image. However, the quality of the H-BTC reconstructed image is improved compared to the original decoded image.

### 2.3. Fast Vector Quantization Based H-BTC Image Reconstruction

The fast vector quantization (FVQ) [24] is an improved version of classical VQ to further speed up the computational time. This approach avoids the closest matching and similarity distance computation for all codeworks in a specific given codebook [6].

**Figure 4:**
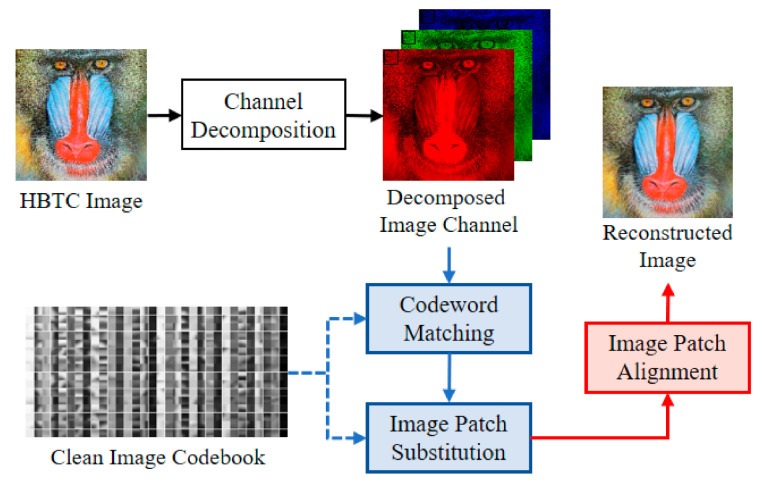
Schematic diagram of fast vector quantization (VQ)-based H-BTC image reconstruction [6].

Figure 4 shows a schematic diagram of the FVQ-based approach [6]. In this scheme, the H-BTC decoded image is firstly divided into several overlapping image patches. These image patches are further matched and replaced with selected codewords from a trained codebook. The VQ or K-means algorithms generate the codebook from a set of clean images.

Let B be a trained codebook of size N, containing several codewords $\left\{C_{1},\;C_{2},\ldots ,C_{N}\right\}$. Each codeword is of n×n. Given p as an image patch from the H-BTC decoded image. This image patch is of the same size with the codeword. The matching process between an image patch p and codewords in codebook B is formulated as:

(15) $$C_{k^{*}}⇐Q\left\{p,\;B\right\},$$

where $Q\left\{\cdot \right\}$ and $C_{k^{*}}$ represent the matching operation and best closest codewords, respectively. This matching process is performed by checking and evaluating the mean value, variance, and norm of the image patch with the offline precomputed of aforementioned values over all codewords. Let $\mu_{p}$, $v_{p}$, and $n_{p}$ be the mean value, variance, and norm value, respectively. The first matching process checks the mean value of uninspected codeword $\mu_{k}$ whether it falls into the following interval:

(16) $$m_{p}-\sqrt{d_{\min}/n^{2}}\leq m_{k}\leq m_{p}+\sqrt{d_{\min}/n^{2}},$$

where $d_{\min}$ is “so far” smallest distortion. If codeword $C_{k}$ satisfies (16), its variance value $v_{k}$ is subsequently checked using the following criterion:

(17) $$v_{p}-\sqrt{d_{\min}}\leq v_{k}\leq v_{p}+\sqrt{d_{\min}}.$$

If the $v_{k}$ value fits on interval (17), the norm value $n_{k}$ is then inspected using:

(18) $$n_{p}-\sqrt{d_{\min}}\leq n_{k}\leq n_{p}+\sqrt{d_{\min}}.$$

If the constraint (18) is satisfied, the codeword $C_{k}$ can be regarded as the best candidate for the closest codeword. Then, the “so far” smallest distortion needs to be updated. The checking process is then continued for all uninspected codewords in the codebook. Subsequently, the image patch p is then simply replaced with codeword $C_{k^{*}}$ as:

(19) $$\tilde{p}⇐C_{k^{*}},$$

where $\tilde{p}$ denotes the replaced image patch. After processing all image patches, the non-alignment H-BTC reconstructed image can be obtained by arranging all image patches as follows:

(20) $$\tilde{o}\left(m,n\right)⇐\cup_{\forall \tilde{p}}\tilde{p}.$$

This process can be viewed as an additional operation over all image patches in the correct position. An alignment operation should be conducted for $\tilde{o}\left(m,n\right)$ to obtain the corrected H-BTC image reconstruction. The image patch alignment can be performed as follows:

(21) $$\tilde{I}\left(m,n\right)⇐\frac{\sum \tilde{o}\left(m,n\right)}{\sum R^{T}\left(m,n\right)R\left(m,n\right)},$$

where $R\left(m,n\right)$ and $\hat{I}$ represent image patch operator and H-BTC reconstructed image, respectively. From this point, the quality of the reconstructed H-BTC image $\tilde{I}\left(m,n\right)$ is increased compared to that of the original H-BTC decoded image.

## 3. Deep Learning Based H-BTC Image Reconstruction

This section’s details present the proposed method for improving the quality of the H-BTC decoded image using a deep learning framework. The proposed method performs image quality enhancement by applying a series of convolutions, downsampling, and upsampling operations. The convolution, downsampling, and upsampling operations play an important role for the proposed method. The convolution operation learns the feature mappings for noise removal, while the downsampling operator effectively reduces the occurred noise. These convolution series effectively extract the important information of the H-BTC decoded image in order to suppress the impulsive noise. Herein, the proposed method inherits the effectiveness of CNN with the residual learning approaches. The proposed method is an extended version of the former scheme published in [14]. The former scheme in [14] mainly focuses on improving the quality of the ODBTC decoded image, while the proposed method aims to increase the quality of H-BTC decoded images, including ODBTC, EDBTC, and DDBTC. Thus, the proposed method can be regarded as a generalized version of the former scheme in [14].

**Figure 5:**
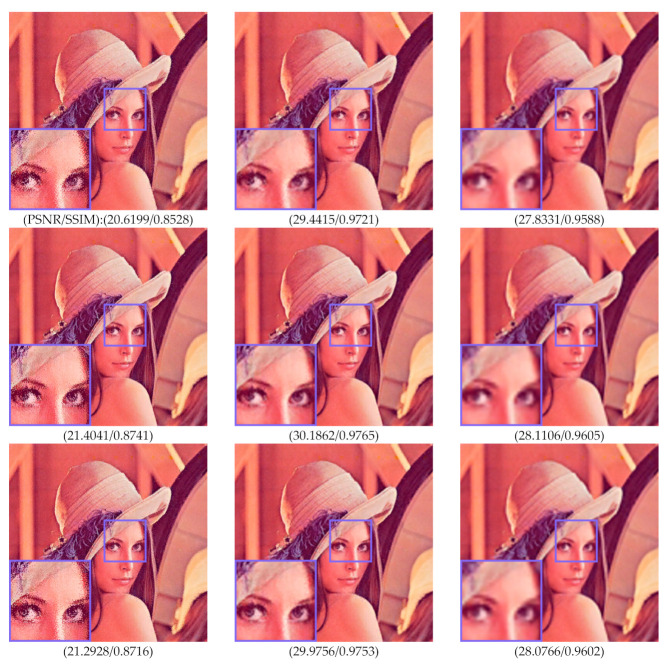
Effects of performing downsampling and upsampling operations on (first to last rows) ODBTC, EDBTC, and DDBTC. The first column is the original decoded image. The second column are reconstructed images after applying downsampling with factor 0.5 and upsampling to the original size, while the third column are images after downsampling operator over factor 0.25 and upsampling to the original size.

The proposed method is motivated from the downsampling and upsampling operation on an image. In some cases, the downsampling operation effectively reduces the occurrence of noise. The quality of the downsampled image is often better compared to the original size of the noisy image. The H-BTC decoded image can be regarded as an image corrupted with the impulsive noise. The first column of Figure 5 shows the decoded image obtained from ODBTC, EDBTC, and DDBTC compressions. The occurred noise is reduced by performing the downsampling operation with factor 0.5 and upsampling back to the original size of the H-BTC decoded images. This result is shown in the second column of Figure 5. Whereas, the third column of Figure 5 is a set of images after performing the downsampling operator with factor 0.25 and upsampling to the original size. The downsampling and upsampling operations suppress the impulsive noise in the H-BTC decoded image effectively.

### 3.1. Residual Concatenated Networks

**Figure 6:**
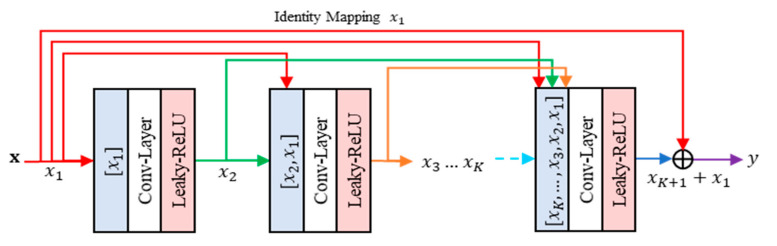
The architecture of residual concatenated networks (RCN).

The proposed method contains the residual leaning part, namely residual concatenated networks (RCN). This RCN consists of multiple convolution layers. This network concatenates each output feature from previous layers, regarded as input features, to the current processed layer. Figure 6 gives an illustration of RCN. Suppose F be an RCN module with input features denoted as x. Let θ be the network parameters. The feed forward process of RCN can be simply formulated as follows:

(22) $$y=F\left(x;\theta \right),$$

where y denotes the output features of RCN. Specifically, the output of each layer while the networks consist of K convolution layers can be written in the concatenated form as follows:

(23) $$x_{k+1}=\varphi (W_{k}*\left[x_{k},x_{k-1},\ldots ,x_{1}\right]+b_{k})\;\forall k=1,2,\ldots ,K,$$

where $x_{1}=x$, $W_{k}$, and $b_{k}$ represent the weights and biases of k-th convolution layer, respectively. The symbols $\left[x_{k},\;x_{k-1},\ldots x_{1}\right]$, *, and φ denote the element-wise concatenation of feature maps, convolution operation of CNN, and Leaky ReLU [8] activation function, respectively. The proposed method exploits the effectiveness of Leaky RELU due to its ability on avoiding the dying RELU while the neuron response is negative. Thus, the proposed method can adapt better learning in the training step.

If the first convolutional layer produces n feature maps, then the k-th convolutional layer will generate a n×k number of feature maps accordingly, except for the K-th layer. The number of features in the final layer should be identical to that of the first convolutional layer. Thus, the final layer could receive the residual information transmitted by shortcut connection as an identity mapping of input features. This process is denoted as follows:

(24) $$y=x_{K+1}+x.$$

The RCN can be regarded as a layer with multiple receptive field configurations by expanding the number of feature maps and integrating the concatenation process.

### 3.2. Residual Networks of Residual Concatenated Networks

**Figure 7:**
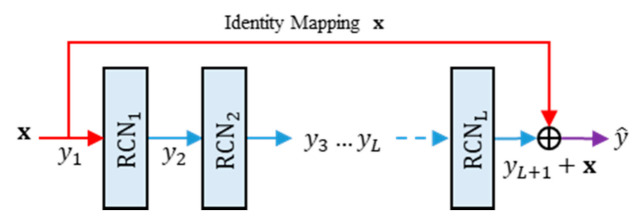
The architecture of residual networks of residual concatenated network (RRCN).

The proposed method also utilizes the residual network of residual concatenated networks (RRCN). Figure 7 depicts a simple illustration of RRCN. This part is a simple residual network with the RCN module as weight layers. By using this network configuration, the information flow can reach the deeper layer of the network easily [7,9]. Let R denote the RRCN operator. Thus, the feed forward process of RRCN can be formally defined as:

(25) $$\hat{y}=R\left(x;\theta \right),$$

where $\hat{y}$ and θ are the output features of RRCN and the RRCN parameters, respectively. Suppose that L be the number of RCN modules in the RRCN part. Thus, the output features of RRCN can be formulated as follows:

(26) $$y_{l+1}=F\left(y_{l};\theta_{l}\right)\;\forall l=1,2,\ldots ,L,$$

(27) $$\hat{y}=y_{L+1}+y_{1},$$

where $\hat{y}$ denotes the output features produced by a RRCN module, with $y_{1}=x$. If the networks is composed from D layers RRCN, then the feed forward process of the network can be written as:

(28) $$z={\hat{y}}_{1}+\sum_{d=1}^{D}R\left({\hat{y}}_{d};\theta_{d}\right)\;\forall d=1,2,\ldots ,D,$$

where z is the networks outputs, with ${\hat{y}}_{1}=x$. The RRCN actually mimics the iterative regularization as similarly performed in the image denoising task, while one observes the following forms:

(29) $$z={\hat{y}}_{1}+R\left({\hat{y}}_{1};\theta_{1}\right)+R\left({\hat{y}}_{2};\theta_{2}\right)+\ldots +R\left({\hat{y}}_{D};\theta_{D}\right),={\hat{y}}_{1}+{\hat{y}}_{2}+{\hat{y}}_{3}+\ldots +{\hat{y}}_{D+1},={\hat{y}}_{1}+\left(y_{L+1}^{\left(1\right)}+{\hat{y}}_{1}\right)+\left(y_{L+1}^{\left(2\right)}+{\hat{y}}_{2}\right)+\ldots +\left(y_{L+1}^{\left(D\right)}+{\hat{y}}_{D}\right).$$

Herein, the RCN module performs the aggregation process. In addition, some important information from preceding layers are retained via shortcut connection as residual information.

### 3.3. Reconstruction Networks

**Figure 8:**
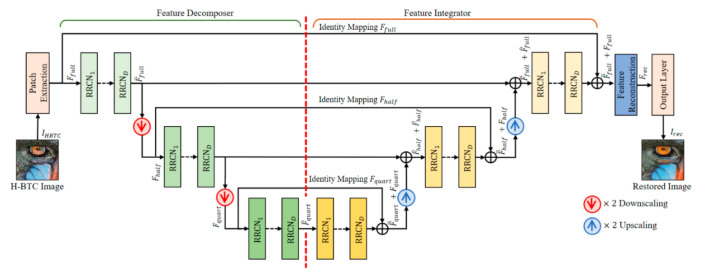
The proposed networks architecture for H-BTC image reconstruction.

This subsection presents the proposed networks for performing the H-BTC image reconstruction. The proposed method is developed and inspired by the effectiveness of wavelet-based approach for suppressing the impulsive noise of the H-BTC decoded image [5] and U-Nets framework [21]. Figure 8 illustrates the proposed networks for H-BTC image reconstruction. The proposed method works on the downscaled version of the H-BTC decoded image. It is based on the fact that the downscaled H-BTC image contains important structural information in comparison to that of the original size of the H-BTC image. The usage of downscaled versions of H-BTC decoded images also significantly reduces the computational overhead required for reconstruction purposes. Compared to the U-Nets framework [21], the proposed method simply utilizes element-wise addition for feature aggregation after downsampling and upsampling operators.

The proposed networks firstly extract some important information of the H-BTC decoded image over various resolutions by performing convolution operation over several layers. Subsequently, the proposed networks reconstruct the feature maps produced by the convolution layers. It receives the H-BTC decoded image, $I_{HBTC}$, as the networks input, and produces the networks output, $I_{rec}$, as the reconstructed image. This process can be formally defined as follows:

(30) $$I_{rec}=\mathrm{RecNet}\left(I_{HBTC};\Theta \right),$$

where $\mathrm{RecNet}\left(\cdot \right)$ and Θ are the operator of reconstruction networks and its corresponding parameters, respectively. Herein, the reconstruction networks consist of five main parts, namely patch extraction layer, feature decomposition layers, feature integration layers, feature reconstruction layers, and output layer. The following are the explanations of each layer.

(1) **Patch Extractor**: This network part owns a single weight layer for performing image patch extraction and representation. Herein, an input image is divided into several overlapping patches. Suppose C denotes the number of image channels. This network part acts as a convolution layer with 32 filters, each of size C×3×3. It generates F feature maps. These feature maps are further processed with nonlinearity mapping activation function, i.e., Leaky ReLU. At the end of the process, this network part yields feature maps of size F×H×W, denoted as $F_{full}$.
(2) **Feature Decomposer**: This network part consists of multiple RRCN and downsampling operators. This network part decomposes and aggregates the extracted feature $F_{full}$ into multiple resolutions of feature maps. The downsampling operations are performed by a convolution layer with two-unit strides. The RRCN in this part employs the RCN modules with dilated convolution. Using this configuration strategy, the receptive field area of networks can be expanded without increasing the number of parameters. This scenario is also motivated by the well-known *algorithme a’trous*, i.e., an algorithm to perform SWT with dilated convolution [10]. The network part produces a feature map ${\bar{F}}_{full}$ of the same size with $F_{full}$. It also generates other maps, i.e., $F_{half}$ and ${\bar{F}}_{half}$, each of half size compared to $F_{full}$. Other maps also yield $F_{quart}$ and ${\bar{F}}_{quart}$, each of quarter size in comparison to that of $F_{full}$.
(3) **Feature Integrator**: This network part has almost similar structure compared to that of the feature decomposer part. The main difference is only on the replacement of the downsampling operator with the upsampling operator. The typical interpolation algorithm, such as bilinear or bicubic, can be selected as an upsampling operator in this network part. The long skip connection in this network part connects the aggregated feature maps from the feature decomposer part and aggregated feature maps from this network part. This long skip connection aims to ease the network training. It can trivially mitigate the modeling of identity mapping in the networks [11]. Yet, the integration process is performed by pixel-wise addition between each channel of feature maps.
(4) **Feature Reconstruction and Output Layers**: Both these layers contain a single weight layer. These two parts perform feature aggregation between the integrated feature maps of ${\overset{=}{F}}_{full}$ and $F_{full}$. After the aggregation operation, we obtain the resulting feature maps denoted as $F_{rec}$. These feature maps are further processed by the output layer to yield the H-BTC reconstructed image, i.e., $I_{rec}$. This $I_{rec}$ is regarded as the improved quality of the H-BTC decoded image with a reduced impulsive noise occurrence.

### 3.4. Loss Function

This subsection shows the loss function of the proposed method for obtaining the optimal H-BTC image reconstruction. Herein, the proposed network architecture performs end-to-end function optimization to achieve the optimal parameters as follows:

(31) $$\hat{\Theta }=\underset{\Theta }{\mathrm{argmin}}\mathrm{RecNet}\left(I_{HBTC};\Theta \right)-I_{GT}{}_{2}^{2},$$

where $\hat{\Theta }$ denotes the optimized network parameters, and $I_{GT}$ is the clean image (ground truth). In simple words, the optimal image reconstruction can be achieved by minimizing the pixel difference between the original image and the reconstructed image produced from the proposed networks.

In this work, we utilize the mean squared error (MSE) as loss function in order to measure the pixel difference between the original and reconstructed image. This loss function is executed during the training process and denoted as follows:

(32) $$L\left(\Theta \right)=\frac{1}{N}\sum_{n=1}^{N}{\left(I_{rec}^{\left(n\right)}-I_{GT}^{\left(n\right)}\right)}^{2},=\frac{1}{N}\sum_{n=1}^{N}{\left(\mathrm{RecNet}\left(I_{HBTC}^{\left(n\right)};\Theta \right)-I_{GT}^{\left(n\right)}\right)}^{2},$$

where N denotes the number of batch sizes for a single iteration. Lower value of this loss function indicates better performance during the training process. Thus, the training procedure is stopped if the magnitude of loss function is small enough or under predetermined maximum iteration. It should be noted that the proposed method is only workable for images of size divisible by 4. However, the proposed method can be applied and generalized for the image over various image sizes. If the image is indivisible by 4, the zero padding can be added to the input image such that it becomes divisible by 4. Yet, we simply extract the image with the original size at the end of the proposed network to obtain an image with the correct size.

The proposed method is mainly designed for reconstructing the color image, i.e., three-dimensional data. However, the proposed method can be further extended for the grayscale image or two-dimensional data without any modifications of the networks. In simple terms, the grayscale image (two-dimensional data) needs to be converted into the color image (three-dimensional data). Each color channel (Red, Green, and Blue channel) is simply set with the grayscale image. Using this strategy, we obtain the color image or three-dimensional data from the grayscale image. Subsequently, this image is further fed into the proposed networks. At the end of the process, the proposed networks produce the image in color space (or three-dimensional data). An additional operation is applied on the output image, i.e., converting back the color image into the grayscale image. We can apply the color image conversion by using formal color space formulation or simply averaging computation over all color spaces. Another way can also be utilized for the grayscale image. The proposed networks can also receive two-dimensional data or grayscale image as input. However, we need to modify the patch extractor layer and output layers.

## 4. Experimental Results

This section reports some extensive experimental results of the proposed method. We firstly describe the image sources including the process of making the H-BTC image dataset. Subsequently, we show the model initialization and experimental setup. The proposed method is visually investigated and compared with the former schemes. At the end of this section, the performance of the proposed method is further compared with some previous H-BTC image reconstruction schemes. We consider the peak signal-to-noise ratio (PSNR) and structural similarity index measure (SSIM) scores [25] to objectively assess the quality of the H-BTC reconstructed image. The PSNR metric evaluates the accuracy of pixel value reconstruction between two images, which is formulated as:

(33) $$\mathrm{PSNR}=20\log_{10}\left(255\right)-10\log_{10}\left(MSE\right),$$

where *MSE* denotes the mean square error value between the reconstructed image and reference image.

On the other hand, the SSIM measures the degree of structural reconstruction between two images. Let $I_{rec}$ and $I_{ref}$ be the reconstructed H-BTC image and the reference image, respectively. The SSIM metric is formally defined as:

(34) $$\mathrm{SSIM}\left(I_{rec},\;I_{ref}\right)=\frac{\left(2\mu_{rec}\mu_{ref}+c_{1}\right)\left(2\sigma_{rec,ref}+c_{2}\right)}{\left(\mu_{rec}^{2}+\mu_{ref}^{2}+c_{1}\right)\left(\sigma_{rec}^{2}+\sigma_{ref}^{2}+c_{2}\right)},$$

where $\mu_{rec}$ and $\sigma_{rec}^{2}$ denote the mean value and variance of H-BTC images, $\mu_{ref}$ and $\sigma_{ref}^{2}$ are the mean value and variance of original image. The value of $\sigma_{rec,ref}$ is the covariance between the H-BTC images and original image. The values of $c_{1}$ and $c_{2}$ are two scalars to stabilize the division operation with weak denominators. The source code of the proposed method will be publicly available on the personal website of the second author.

### 4.1. Experimental Setup

We firstly describe the experimental setup for the proposed method. The proposed method needs three image datasets, regarded as the training, validation, and testing image sets. For the training set, we use a set of images from the DIV2K image dataset [26]. Herein, each image is divided into non-overlapping image patches of size 128×128 pixels. Thus, we obtain 167,235 image patches for the training of the proposed H-BTC reconstruction network. In the training process, the proposed method requires a paired image, i.e., the H-BTC compressed image and its corresponding original image. For each training image patch, we simply perform the ODBTC, EDBTC, and DDBTC image compression to create the H-BTC compressed image from the original image patch. The size of the H-BTC compression is set as 8×8 and 16×16. The proposed method is workable on any arbitrary H-BTC image size, not only 8×8 and 16×16. We can simply perform image reconstruction using the proposed method with any arbitrary H-BTC block sizes. Yet, we can feed a set of H-BTC compressed and original image patches for the training purpose.

Despite using the training image set, we also need another image sets, i.e., validation set, in the training process. This validation set is to monitor the reconstruction performance during the training process. Herein, we use the downsampled version of the validation set (low resolution with bicubic interpolation of scale ×4) from DIV2K [26]. There are six images for the validation set. We perform a similar process as used in the training set for this validation set. The BSD100 [27], 24 images from Kodak [28] image dataset, and 16 images test from [5,6] are involved in the experiment as testing sets. They contain some broadly used images such as Lenna, Baboon, airplane, peppers, etc. All those image datasets consist of natural scenes. In addition, we also observe the proposed method performance under the URBAN100 [27] image dataset. This dataset is very challenging since it contains some urban scenes with rich details in various frequency bands. It should be noted that the dithering process on H-BTC image compression usually destroys the image details and lines. In our experimental activity, the images for validation and testing purpose are excluded from the training set.

### 4.2. Networks Training and Model Initialization

The network weights for the proposed method are initialized using He’s method [12]. Whereas, all biases in the proposed networks are firstly set as zero over all layers on the initial stage. The proposed networks employ the stride of size 1 for all kernels, except some kernels for the downsampling operation. The proposed method simply sets the stride with size 2 for the kernel of downsampling. In addition, the proposed scheme utilizes the kernels with size 3 × 3 over all layers. Herein, we utilize the Adam algorithm [13] for performing the optimization task with $\beta_{1}=0.9$, $\beta_{2}=0.999$, and $\epsilon =1\times 10^{-8}$. The training process is performed on 20 epochs (roughly 200.000 iterations), with batch normalization of size 16 images. The learning rate is set $1\times 10^{-4}$ for the first 10 epoch and set into $1\times 10^{-5}$ for the rest training of epoch. We use two RRCN modules, i.e., D=2 in each network part. Each module contains three RCN modules, i.e., L=3. Yet, each RCN modules consists of three convolutional layers, i.e., K=3. The constant of all Leaky ReLU is set to 0.0001. All experiments were conducted under the Pytorch framework [29] and Python 3. The experimental environment was set on a computer with AMD Ryzen™ Threadripper 1950X 3.4GHz CPU and Nvidia GTX 1080 ti GPU. It approximately requires about 20 h for performing the training process of the proposed method.

**Figure 9:**
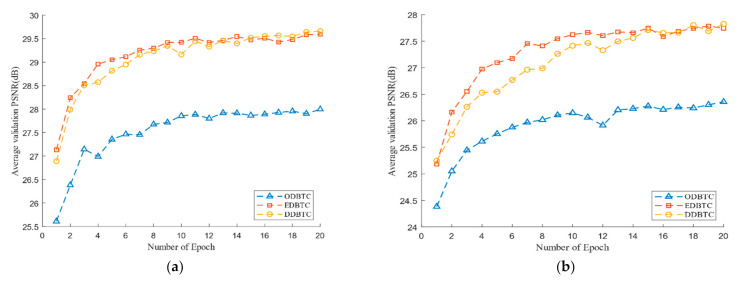
The performance of the proposed method in terms of average peak signal-to-noise (PSNR) values during the training process over various H-BTC image block sizes: (**a**) 8 × 8, (**b**) 16 × 16.

Figure 9 shows the performance of the proposed method during the training process over the H-BTC image block size 8×8 and 16×16. Herein, we use the average PSNR to measure the performance over several epochs. The PSNR is computed for all images in the validation set. From Figure 9, the number of epoch 20 is enough to obtain near-optimum networks parameters for the proposed method. During the training process, the ODBTC image compression is relatively hard to achieve the optimum solution compared to the other H-BTC methods. Since the impulsive noise produced by ODBTC is more randomized and perceived, it causes difficulty to the proposed networks to obtain an optimum solution in a limited number of epochs.

**Figure 10:**
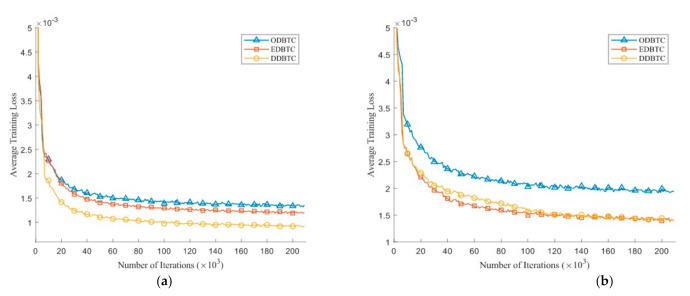
The average training loss of the proposed method over various H-BTC image blocks: (**a**) 8×8, and (**b**) 16×16.

**Figure 11:**
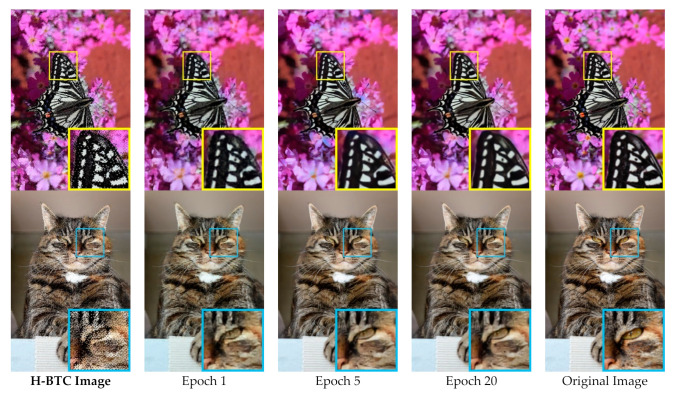
Image quality improvement produced by the proposed networks during the training process on the validation set.

**Figure 12:**
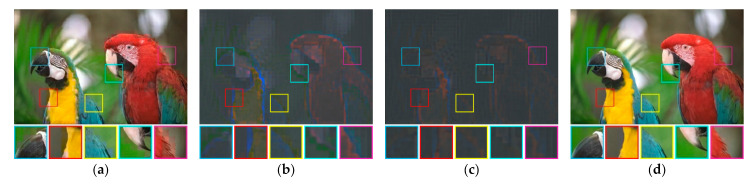
An example of image block alignment performed by the proposed networks: (**a**) ${\overset{=}{F}}_{full}$, (**b**) ${\overset{=}{F}}_{\mathrm{half}}$, (**c**) ${\overset{=}{F}}_{\mathrm{quart}}$, and (**d**) the final reconstruction image obtained by combining all those features.

Figure 10 depicts the average training loss during the training process on the validation set over various number of iterations. Herein, we set the H-BTC image block as 8×8 and 16×16. After several iterations, the average training loss does not significantly improve. It indicates that the proposed method almost finds an optimum solution. Similar to the previous finding, the impulsive noise on ODBTC is also hard to be suppressed. The effect of different epochs on visual quality of the H-BTC reconstructed image is shown in Figure 11. In this experiment, we show the quality improvement produced by the proposed method on an image from the validation set while the parameters of networks are from epoch 1, 5, and 20. From this figure, we can clearly see that the quality of the H-BTC reconstructed image is progressively improved while the number of epochs goes. Increasing the number of epochs may produce better quality on the H-BTC reconstructed image. In addition, we give an example of image block alignment performed by the proposed method as shown in Figure 12. As shown in this figure, the proposed method effectively increases the quality of the H-BTC decoded image by combining all image features.

### 4.3. Visual Investigation

**Figure 13:**
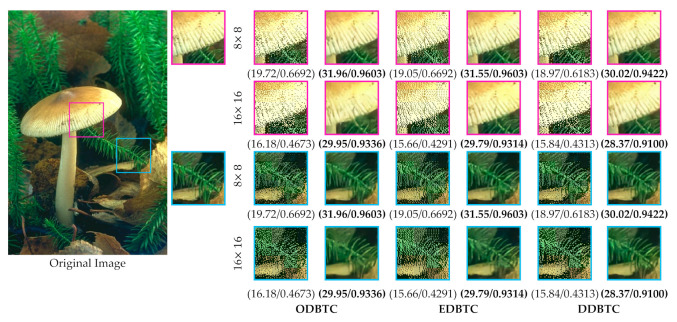
Performance comparisons over various H-BTC methods on an image 208001 from the BSD100 dataset.

**Figure 14:**
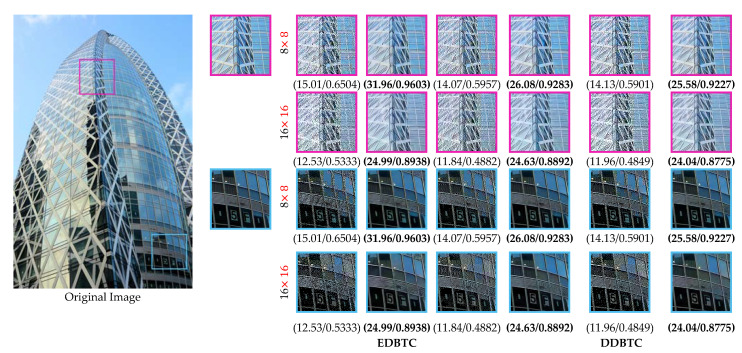
Performance comparisons over various H-BTC methods on an image img_039 from the URBAN100 dataset.

This section reports the performance of the proposed method based on the visual investigation. We firstly investigate the performance of proposed method over various H-BTC image compression methods, i.e., ODBTC, EDBTC, and DDBTC. The image block for H-BTC is set as 8×8 and 16×16. In this experiment, we train the proposed networks with the training set, and then validate the performance using the training set. Yet, we overlook the performance by applying the optimum networks parameters on the testing set. Figure 13 and Figure 14 show the visual comparisons for the proposed networks over various H-BTC methods. The quality of H-BTC reconstructed image yielded by our proposed method is better than the original H-BTC decoded image. It clearly reveals that the proposed method can remove the occurrence of impulsive noise on the H-BTC decoded image. In addition, the proposed method is capable of removing the blocking artifact that appeared on the H-BTC decoded image. Yet, the proposed method effectively refines the damaged image details and lines caused by digital halftoning.

**Figure 15:**
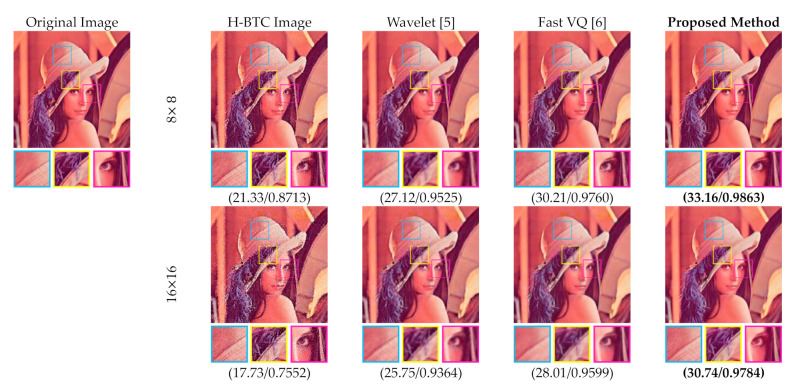
Visual comparisons between the proposed method and former schemes on reconstructing the DDBTC decoded image over the Lena image.

**Figure 16:**
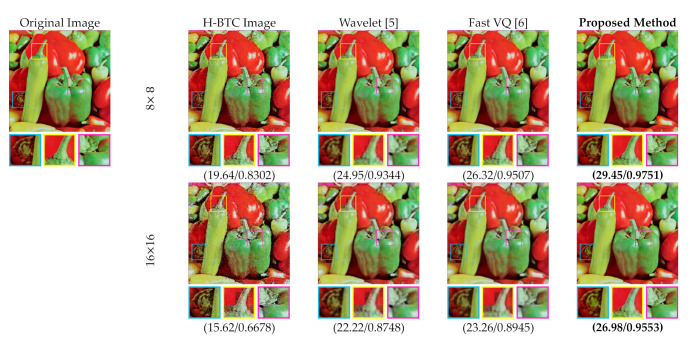
Visual comparisons between the proposed method and former schemes on reconstructing the DDBTC decoded image over the Peppers image.

Figure 15 and Figure 16 display the performance comparisons between the proposed method and former schemes in the H-BTC image reconstruction task. Herein, we only consider the DDBTC compression with the block size 8×8. We visually compared the former wavelet-based and FVQ-based approaches. As displayed in this figure, the proposed method produces better image quality in comparison with the former competing schemes as indicated with higher SSIM and PSNR scores. The proposed method also outperforms the other techniques based on the reconstructed image quality. One may conclude that the proposed method is capable of increasing the quality of the H-BTC decoded image.

### 4.4. Performance Comparisons

This subsection reports the performance comparison between the proposed method and former schemes in terms of objective measurements. In this experiment, the optimum parameters of proposed networks are obtained from the training process as described before. These parameters are then applied to the testing set. Herein, we consider all images from SIPI, Kodak, BSD100, and Urban100 as testing images. For each image, we perform H-BTC image compression under the image block 8×8 and 16×16. Subsequently, each decoded image is processed by the proposed method with the optimum parameters. The quality of reconstructed image is then measured using two metrics, i.e., SSIM and PSNR. We firstly compare the performance of the proposed method and former schemes in terms of average PSNR value. Herein, we make comparisons against the traditional approaches for H-BTC image reconstruction such as the wavelet-based method [5] and FVQ [6] scheme. In addition, the comparison is also conducted with the former deep learning-based approaches such as in the residual network [15,16] and symmetric skip CNN [17]. The methods in [15,16] inherit the superiority of the residual networks for performing the image denoising. These methods are also very suitable for H-BTC image reconstruction under the similarity of the ability of noise suppression. Meanwhile, the method in [17] performs H-BTC image reconstruction by involving the symmetric skip CNN framework. To make a fair comparison, we simply set the number of layers and feature maps for the former schemes [15,16,17] as identical to the proposed method. Table 1 shows the performance comparisons between the proposed method and former schemes in terms of average PSNR value. Whereas, Table 2 summarizes the performance comparison between the proposed method and former existing schemes in terms of the average SSIM score. These two tables explicitly tell that the proposed method outperforms the other competing schemes in the H-BTC reconstruction task. It is worthy noted that the proposed method yields better results with significant margin in comparison to the various traditional or handcrafted H-BTC compression methods. In addition, the proposed method also yields better performance in comparisons to the former deep learning-based approaches. The proposed method is a good candidate in the H-BTC image reconstruction task. The proposed method can be extended and applied for image compression, vehicle verification [30], and secret sharing [31,32,33].

## 5. Conclusions and Future Works

A deep learning approach for H-BTC image reconstruction has been presented in this paper. The H-BTC aims to perform image compression with simple technique. The proposed method inherits the effectiveness of CNN and residual learning frameworks to perform the reconstruction process. It is constructed from RCN and RRCN modules to suppress the impulsive noise and reduce the blocking artifacts of the H-BTC decoded image. The proposed method presented in this paper can be regarded as a post-processing step in the H-BTC image compression technique. As documented in the Experimental Results Section, the proposed method offers better quality on the H-BTC reconstructed image compared to the former schemes. In the real application, the proposed method can be applied on the post-processing of image compression while the decoded image requires the noise suppression or quality enhancement. The proposed method works well on improving the quality of the decoded image obtained from various image compression techniques. The decoded image can be simply fed into the proposed framework and produce the enhanced image. In addition, the proposed method can also be used for improving the quality of the H-BTC decoded image for image retrieval, watermarking, secret sharing, and the other image processing and computer vision applications.

To further improve the performance of the proposed method, the number of layers can be added to the proposed networks in the H-BTC image reconstruction task. The different activation functions can also be investigated to increase the learning ability of the proposed networks. The proposed networks can also use different optimizers to increase its performance. The convolutional operation in the proposed method can also be modified by incorporating the non-local self-similarity, graph convolutional approach, nearest neighbor information, non-local recurrent framework, and so forth. These aforementioned approaches may improve the convolutional ability to capture the non-local information to increase the learning ability and performance. In addition, the adversarial learning can be injected to the proposed networks. The adversarial networks can effectively learn the occurrence of impulsive noise and suppress the detected noise in encoder–decoder based learning. The residual learning with adversarial networks may yield better performance in H-BTC image reconstruction. These alternatives are our future works and suggestions.

## Figures and Tables

**Table 1:** Performance comparison between the proposed method and former schemes in terms of PSNR value.

**ODBTC**							
**Datasets**	**Block Size**	**Decoded**	**Wavelet [5]**	**Fast VQ [6]**	**Residual Network [15,16]**	**Symmetric Skip CNN [17]**	**Proposed**
SIPI	8×8	19.73	25.12	27.34	28.11	29.31	**29.90**
	16×16	16.58	23.57	25.06	26.90	27.22	**27.53**
Kodak	8×8	19.25	24.55	27.48	28.91	29.45	**30.58**
	16×16	16.16	23.74	25.68	27.89	28.67	**29.00**
BSD100	8×8	17.94	23.80	26.55	28.09	28.33	**28.79**
	16×16	15.06	23.14	24.97	26.88	26.98	**27.51**
Urban100	8×8	16.15	20.89	23.93	27.34	27.77	**28.26**
	16×16	13.48	20.30	22.27	25.89	26.31	**26.80**
**EDBTC**							
**Datasets**	**Block size**	**Decoded**	**Wavelet [5]**	**Fast VQ [6]**	**Residual Network [15,16]**	**Symmetric Skip CNN [17]**	**Proposed**
SIPI	8×8	19.80	25.12	27.44	29.39	29.77	**30.78**
	16×16	16.40	23.52	24.94	27.71	28.03	**28.46**
Kodak	8×8	19.28	24.61	27.83	30.44	31.78	**32.03**
	16×16	15.98	23.80	25.64	29.33	29.78	**30.40**
BSD100	8×8	17.95	23.85	26.91	28.98	29.78	**30.41**
	16×16	14.88	23.22	24.94	28.45	29.00	**29.11**
Urban100	8×8	16.21	20.90	24.17	28.65	28.91	**29.20**
	16×16	13.35	20.33	22.22	26.01	26.77	**27.61**
**DDBTC**							
**Datasets**	**Block size**	**Decoded**	**Wavelet [5]**	**Fast VQ [6]**	**Residual Network [15,16]**	**Symmetric Skip CNN [17]**	**Proposed**
SIPI	8×8	2.037	25.00	27.68	28.89	29.78	**30.79**
	16×16	16.89	23.42	25.25	27.63	28.01	**28.13**
Kodak	8×8	20.02	24.48	28.31	30.33	31.78	**32.26**
	16×16	16.53	23.68	26.04	29.11	30.37	**30.48**
BSD100	8×8	18.75	23.72	27.50	28.88	29.79	**30.7**4
	16×16	15.47	23.10	25.40	28.01	28.99	**29.19**
Urban100	8×8	17.04	20.83	24.71	28.64	28.91	**29.54**
	16×16	13.96	20.24	22.63	27.12	27.78	**27.90**

**Table 2:** Performance Comparison between the Proposed Method and Former Schemes in Terms of SSIM Score.

**ODBTC**							
**Datasets**	**Block Size**	**Decoded**	**Wavelet [5]**	**Fast VQ [6]**	**Residual Network [15,16]**	**Symmetric Skip CNN [17]**	**Proposed**
SIPI	8×8	0.7188	0.8632	0.9132	0.9391	0.9399	**0.9462**
	16×16	0.5677	0.8118	0.8575	0.8911	0.9102	**0.9186**
Kodak	8×8	0.6425	0.8026	0.8921	0.9220	0.9378	**0.9414**
	16×16	0.4820	0.7624	0.8299	0.8998	0.9002	**0.9155**
BSD100	8×8	0.5967	0.7778	0.8698	0.8978	0.9100	**0.9193**
	16×16	0.4373	0.7333	0.8008	0.8698	0.8709	**0.8891**
Urban100	8×8	0.5940	0.7124	0.8266	0.9301	0.9299	**0.9315**
	16×16	0.4466	0.6605	0.7443	0.8760	0.8923	**0.9006**
**EDBTC**							
**Datasets**	**Block size**	**Decoded**	**Wavelet [5]**	**Fast VQ [6]**	**Residual Network [15,16]**	**Symmetric Skip CNN [17]**	**Proposed**
SIPI	8×8	0.7242	0.8639	0.9153	0.9312	0.9457	**0.9551**
	16×16	0.5669	0.8138	0.8539	0.8991	0.9129	**0.9291**
Kodak	8×8	0.6512	0.8055	0.9005	0.9413	0.9550	**0.9569**
	16×16	0.4818	0.7705	0.8322	0.9221	0.9331	**0.9349**
BSD100	8×8	0.6034	0.7781	0.8802	0.9378	0.9399	**0.9415**
	16×16	0.4374	0.7436	0.8044	0.9000	0.9167	**0.9173**
Urban100	8×8	0.6048	0.7145	0.8348	0.9232	0.9389	**0.9433**
	16×16	0.4538	0.6686	0.7455	0.8975	0.9099	**0.9124**
**DDBTC**							
**Datasets**	**Block size**	**Decoded**	**Wavelet [5]**	**Fast VQ [6]**	**Residual Network [15,16]**	**Symmetric Skip CNN [17]**	**Proposed**
SIPI	8×8	0.7530	0.8619	0.9216	0.9391	0.9478	**0.9552**
	16×16	0.5932	0.8101	0.8626	0.9090	0.9199	**0.9287**
Kodak	8×8	0.6940	0.8016	0.9129	0.9378	0.9457	**0.9594**
	16×16	0.5191	0.7622	0.8427	0.9229	0.9301	**0.9385**
BSD100	8×8	0.6551	0.7741	0.8966	0.9331	0.9441	**0.9474**
	16×16	0.4797	0.7345	0.8162	0.9032	0.9210	**0.9220**
Urban100	8×8	0.6543	0.7140	0.8541	0.9234	0.9299	**0.9481**
	16×16	0.4906	0.6612	0.7607	0.9163	0.9200	**0.9210**

## Data Availability

This study did not report any data.
